# Learning English as a Foreign Language Writing Skills in Collaborative Settings: A Cognitive Load Perspective

**DOI:** 10.3389/fpsyg.2022.932291

**Published:** 2022-06-30

**Authors:** Dayu Jiang, Slava Kalyuga

**Affiliations:** ^1^School of Foreign Languages and Literature, Wuhan University, Wuhan, China; ^2^School of Education, University of New South Wales, Sydney, NSW, Australia

**Keywords:** collective working memory effect, cognitive load theory, collaborative writing, teaching English as a foreign language, process-genre approach

## Abstract

Learning to write in a foreign language is a complex cognitive process. The process-genre approach is a common instructional practice adopted by language teachers to develop learners’ writing abilities. However, the interacting elements of procedural knowledge, linguistic knowledge, and generic knowledge in this approach may exceed the capacity of an individual learner’s working memory, thus actually hindering the acquisition of writing skills. According to the collective working memory effect, it was hypothesized that teaching writing skills of English as a foreign language by adopting a process-genre approach in collaborative conditions could lead to better writing performance, lower cognitive load, and higher instructional efficiency. The reported experiment compared learning writing skills of English as a foreign language in individual and collaborative instructional conditions from a cognitive load perspective, a rarely adopted approach in this field. The results indicated that the collaborative instructional condition was more effective and efficient than the individual instructional condition in improving the quality of written products as well as in optimizing the cognitive (working memory) load experienced by the learners. Measures of cognitive load were used to support the cognitive load theory’s interpretation of the results, which is the unique contribution of this research study to the field.

## Introduction

Learning to write in a foreign language is a complex problem-solving process, requiring not only a range of skills from writing English letters to composing complete essays but also the ability to make claims and provide appropriate supporting details (Kirkland and Saunders, 1991; Bruning and Horn, 2010; Howell et al., 2018). Students need to develop the skills of generating, organizing, and refining ideas by being involved in complex activities, such as brainstorming, discussing, outlining, drafting, monitoring, and revising (Raimes, 1992; Hyland, 2003a). Cognitive load theory aims at designing effective instructional materials and procedures to facilitate learners’ acquisition of complex knowledge and skills based on the mechanisms of human cognitive architecture (Van Merriënboer and Sweller, 2005; Sweller et al., 2011). According to this theory, learners can build new knowledge about writing processes not only with the help of explicit formal instruction or through personal reading but also using problem solving via individual or collaborative efforts (through personal introspection or pair/group discussions).

The collective working memory effect in cognitive load theory refers to the working memory space created by communicating and coordinating knowledge by each collaborator (Kirschner et al., 2011, 2018; Sweller et al., 2011). An individual who studies alone processes all the interacting elements of the instructional material in his or her working memory. By contrast, under a collaborative learning condition, all the interactive elements can be distributed among the working memories of group members. This effect allows a better understanding of cognitive processes in collaborative learning environments and the conditions under which such environments provide more efficient instructional options.

However, to our best knowledge, differences between the effectiveness of individual and collaborative instructional approaches in learning writing skills of English as a foreign language from a cognitive load perspective have never been investigated (Kirschner et al., 2011, 2018). Moreover, despite that collaborative writing as a teaching strategy has been actively implemented in foreign language classrooms since the 1990s (McDonough, 2004; Shehadeh, 2011), the issue of how developing writing skills in collaborative settings impact learners’ cognitive characteristics has not been investigated extensively. In addition, more empirical research should be done to examine how learners in collaborative learning conditions would perform on individual writing tasks rather than on co-authoring tasks in the post-intervention phase (Storch, 2005; Chen, 2019). Accordingly, the experimental study reported in this paper was conducted in an attempt to fill these gaps.

## Models and Approaches to Teaching Writing Skills

### Cognitive Model of Writing Processes

Writing involves a range of cognitive activities. Flower and Hayes (1981) proposed a cognitive model of writing processes, which regarded writing as a decision-making process, consisting of a range of cognitive activities orchestrated in cyclical or recursive rather than linear orders (Racelis and Matsuda, 2013). Flower and Hayes (1981) argued that a writing process “involves three major elements which are reflected in the three units of the model: the task environment, the writer’s long-term memory, and the writing process” (p. 369). This cognitive model generally corresponds to the three phases of writing: planning, translating, and revising phases. The three cognitive processes do not necessarily appear in a linear order but can happen at any moment in the writing process (Berninger et al., 1996; Baaijen and Galbraith, 2018, p. 196). Jones (2014) highlighted that the cognitive model of writing processes emphasized the functions of planning (i.e., generating ideas) and translating ideas into texts. Even though Flower and Hayes (1981) stressed that the three types of cognitive activities were recursive, they did not identify the “distinctions involving the temporal dimensions (before, during, or after translation) and spatial dimensions on which the planning and reviewing/revising processes operate (whole text or a portion of it)” (Berninger et al., 1996, p. 198). The distinctions are of great significance to instructions as an awareness of stages or phases in writing could help learners internalize the phases of writing, which was evidenced in Jones’ (2014) study that some of the participants were not fully aware of making distinctions between planning and translating while others were struggled with how to organize ideas in the writing process. It can be assumed that explicit instruction in planning and organizing ideas in the pre-writing stage could improve writing quality. Orchestrating the cognitive activities into stages or phases in this study attempted to actualize these abstract activities for instructional purposes. However, as Bizzell (1982) and Atkinson (2003) noted, this post-cognitivist approach to writing may neglect the genre nature of writings—shared features of texts shaped through social conventions. Therefore, it is of equal significance to teach genre knowledge when adopting the cognitive model of writing processes in teaching writing skills.

### Approaches to Teaching Writing Skills

The genre approach and process approach to teaching writing skills have been used extensively to promote learners’ abilities to write in English (Hyland, 2003a,b; Muncie, 2009; Keen, 2020). The process-based approach in writing instruction, which was introduced in the 1980s, usually consists of four stages: prewriting, writing, revising, and editing (Tribble, 1996). Participants in Keen’s (2020) study adopted a process approach to learning skills: discussing topics in small groups, writing ideas about the topic, writing first drafts, carrying out peer reviews, writing second drafts, and sharing their accounts with the whole class. It was found that the participants developed a sense of ownership and learned how to write more effectively. Even though Keen (2020) used young learners of English as a first language as research subjects, he identified the beneficial role of procedural learning in cultivating students’ writing abilities. However, it should be noted that such approaches demonstrate “how some writers write, they do not reveal why they make certain linguistic and rhetorical choices” (Hyland, 2003b, p. 19), as the process-based approach “is seen as predominantly to do with linguistic skills such as planning and drafting, and there is much less emphasis on linguistic knowledge” (Badger and White, 2000, p. 154). In a response, (Hyland, 2003b,^2008^) put forward a genre-based approach to teach writing skills, in which genre is conceptualized as “a term for grouping texts together, representing how writers typically use language to respond to recurring situations” (p. 544). The genre-based approach emphasizes explicit instructions for communicative purposes, key language features, and structural patterns.

Graham and Sandmel (2011) advised that “advocates of process writing instruction integrate other effective writing practices into this approach” (p. 405). Researchers (e.g., Flowerdew, 1993; Badger and White, 2000) have endeavored to integrate the process-approach and genre-based approach in teaching writing skills of English as a foreign language as the two approaches could be mutually complementary (Raimes, 1991; Badger and White, 2000; Racelis and Matsuda, 2013; Deng et al., 2014; Huang and Zhang, 2020; Jiang et al., 2021; Rahimi and Zhang, 2021). For example, Flowerdew (1993) introduced a process consisting of six types of activities to explicitly teach the process of learning specific genres. Badger and White (2000) proposed the process-genre approach to teaching writing skills, which consists of several stages starting from understanding a situation to completing a draft. By process-genre approach, Badger and White (2000) emphasized the significant roles of language skills, situational knowledge, and processes in cultivating writing abilities. Learning to write also means learning the techniques of self-regulating cognitive activities and procedures. Students who learn how to regulate the writing procedures collaboratively could transfer the knowledge when writing independently (Teng, 2020).

### Learning English Writing Skills Through Collaboration

Taking a social stance, a process-genre approach to teaching writing skills encourages interactions and collaborations, which involves some kinds of collaborative activities such as “modeling, eliciting, supporting, probing, and suggesting alternatives or extension” to a learner’s initial attempts (Wette, 2017, p. 72). Dillenbourg (1999) and Prince (2004) defined collaborative learning as an instructional method through which students work together in small groups to pursue common learning or writing goals. Although collaborative learning, in general, has a long history of research, learning writing skills through collaboration was not actively implemented in foreign language classrooms until the late 1990s (McDonough, 2004). Learning writing skills through collaboration, with a primary aim of learning curricular content, focuses on both deconstruction and construction processes (Karnes et al., 1997). Granado-Peinado et al. (2019) found that participants who received collaborative practice and explicit instructions about writing synthesis identified more proportions of arguments and higher levels of integration of different sources than those in the collaborative practice conditions without instructions about writing synthesis. However, their research showed that providing collaboration opportunities does not sufficiently warrant effective learning, which also needs not only guides about how to collaborate but also explicit instructions about learning tasks. Accordingly, Teng (2020) investigated the effect of collaboratively modeling text structure and explicitly teaching self-regulated strategies on younger English learners’ abilities to write summarizations and essays. After 1-month intervention, it was found that participants who adopted self-regulated strategies and collaboratively modeled text structures demonstrated better performance than the participants in the control group in terms of the three measurements. It should be noted that the available research studies have reported mixed results about whether learning writing skills through collaborations could effectively improve the quality of written products or not (McDonough, 2004; McDonough and De Vleeschauwer, 2019; Matos, 2021). For example, some studies (e.g., Storch, 2005; Fernández Dobao, 2012; Hsu and Lo, 2018) indicated that texts written by collaborative learners were more grammatically accurate than those by individual ones. However, it has also been reported that learners in the individual learning conditions produced more syntactically complex text than collaborative learners (McDonough et al., 2018). The divergent findings in the collaborative learning of writing skills can be related to the following three issues: the lack of explicit collaborative tasks in the learning phases, not considering cognitive aspects in the experimental designs, and not evaluating individual writing outcomes. Accordingly, Kirschner et al. (2009) recommended that research in collaborative learning should directly measure learning outcomes in a test condition, focus on one aspect of the learning goals at a time, and investigate the performance of individual learners instead of the group as a whole. They also advocated that research studies need to consider human cognitive architecture to better understand and compare individual and collaborative learning. In addition, (Berninger et al., 1996) noted that “working memory, and not only long-term memory, is involved in writing development” (p. 199), as the cognitive activities in relation to the task environment and writing process should be carried out in working memory.

## Cognitive Load Theory

Cognitive load theory aims at designing effective instructional materials and procedures to optimize learner cognitive resources in the process of acquiring complex knowledge structures (Sweller, 2010; Sweller et al., 2011). Cognitive load refers to the working memory resources needed for completing a particular learning task. Theoretically, learners may experience two types of cognitive load: intrinsic cognitive load and extraneous cognitive load (Van Merriënboer and Sweller, 2005; Sweller et al., 2011). Intrinsic cognitive load is defined as the working memory resources demanded by the innate complexity of information that a learner must learn (Sweller, 2010). Extraneous cognitive load, conceptualized as the working memory load that is unnecessary and extrinsic to instructional goals, is generated by the presentation manner and structure of the instructional material (Van Merriënboer and Sweller, 2005; Sweller et al., 2011).

The level of cognitive load experienced by the learners is determined by the level of element interactivity which refers to the degree to which information elements or components of a learning task should be processed simultaneously for meaningful learning (Sweller et al., 2011). For example, learning new vocabularies in a list can be considered as low in element interactivity, as individual vocabularies can be acquired without reference to other information in the list. By contrast, most writing tasks have high levels of element interactivity, as the writing process involves a relatively large number of interconnected elements of information, as well as cognitive, metacognitive, and socio-affective activities (Negari, 2011).

The levels of cognitive load that learners experience can be measured by subjective rating scales of effort, a simple and reliable instrument first adopted by Paas (1992). In this type of rating method, learners were asked to recall, reflect, and report the level of mental effort during their previous learning after they completed instructional activities. Even though subjective rating scales were capable of measuring the overall cognitive load, researchers also needed information about the levels of particular types of cognitive load that learners experience (Paas et al., 2003; DeLeeuw and Mayer, 2008). Leppink et al. (2013) proposed a more recent version of subjective rating scales: three items on intrinsic cognitive load, three items on extraneous cognitive load, and four items on germane cognitive load. However, the results of confirmatory factor analysis in Jiang and Kalyuga’s (2020) study showed that the two-factor (intrinsic and extraneous) model was an acceptable fit. Therefore, the cognitive load rating questionnaire in this study, which was developed on the basis of Leppink et al.’s (2013) version, adopted the two-factor model.

Cognitive load ratings are frequently combined with learning performance measures to calculate the relative instructional efficiency for different learning environments. Instructional efficiency in this study was calculated using Paas and van Merriënboer’s (1993) formula *E* = (*P-R*)/√*2*, in which *E* stands for efficiency, *P* for performance z-score, and *R* for cognitive load rating z-score. In this study, the average of intrinsic cognitive load and extraneous cognitive load ratings were used to calculate the cognitive load z-score. According to this formula, higher values of instructional efficiency are achieved in situations where learning performance is high and cognitive load is low; lower values of instructional efficiency occur under conditions where learning performance is low and cognitive load is high.

### Collective Working Memory Effect

Cognitive load theory considers a social interaction situation as a collective working memory system and extends the instructional focus from individual learning to collaborative learning. A collective working memory system can be developed from individual cognitive systems through collaboration, coordination, and communication. The collective working memory effect happens when learners acquire knowledge more effectively and efficiently through collaborating with others than through learning individually (Sweller et al., 2011). The collective working memory space constituted by multiple working memories has a larger capacity and longer duration than any of the constituents in individual working memories. This concept was supported by Dillenbourg (1999) who argued that in the collaborative conditions, “the horizontal division of labor into, for instance, task-level and strategy-level tasks, reduces the amounts of processing performed by each individual” (p. 10). Villarreal and Gil-Sarratea (2019) found that the texts produced by pairs were more accurate and grammatically complex than those by individual learners. They attributed the difference partially to collective scaffolding.

Collective working memory refers to the working memory space created by communicating and coordinating knowledge by each collaborator (Kirschner et al., 2018). An individual who studies alone processes all the interacting elements of the instructional material in his or her working memory. By contrast, under a collaborative learning condition, all the interactive elements can be distributed among the working memories of group members. The multiple working memories constitute a collective working memory space that has a larger capacity and longer duration than individual working memory. As a result, an individual learner in the collaborative instructional condition may experience lower levels of the cognitive load than a learner who studies alone. The collective working memory effect, a recently developed cognitive load theory effect, occurs when learners learn better through collaborating with other learners than through learning alone (Sweller et al., 2011). This effect assumes that “students working in groups have more processing capacity than students working individually” (Janssen et al., 2010, p. 139). Even though interacting with group members in the collaborative learning condition may generate extraneous cognitive load, the interactive process should be beneficial as elaborating and eliciting could result in forming more advanced knowledge (Dillenbourg, 1999).

Under the individual learning condition, all the interacting elements of the learning task are processed in the individual learner’s working memory. By contrast, learners who collaborate with others in their learning distribute all the interactive elements among the working memories of group members. Consequently, a collaborator would experience lower levels of the cognitive load than an individual learner. This assumption was supported by Zhang et al. (2011), who compared the effectiveness of collaborative and individual instructional approaches in learning the complex tasks of designing web pages. They found that the participants in the collaborative learning condition demonstrated better performance and experienced a lower level of the cognitive load than the individual learners.

Task complexity or element interactivity can influence the effectiveness of collaborative learning. For simple learning tasks, individual learning is expected to be more effective and efficient, as the transaction costs associated with sharing knowledge and coordinating communication will nullify the benefits offered by collaborative learning. By contrast, for complex tasks, the benefits offered by the collective working memory could be higher than the transaction costs, thus fostering efficient learning. Kirschner et al. (2009) found that individual learners performed better in remembering biological knowledge (simple tasks) than learners in collaborative conditions, whereas collaborative learners performed better in transferring the skills to solving similar problems (complex tasks) than individual learners. Similar findings were reported by Kirschner et al. (2011) who found that learning low-complexity biological tasks individually was more effective and efficient while learning high-complexity tasks benefited more from the collaborative approach.

## Experimental Study

Learning writing skills of English as a foreign language has long been regarded as a complex process that usually generates a heavy cognitive load (Vanderberg and Swanson, 2007; Kellogg, 2008). Based on the review of literature on cognitive load theory and writing learning, the study was conducted to examine the following research hypotheses:

(1) Participants taught through the process-genre approach in the collaborative learning condition would demonstrate better individual writing performances than participants in the individual learning condition.
(2) Participants taught through the process-genre approach in the collaborative learning condition would experience lower levels of the cognitive load than participants in the individual learning condition.

The reported experiment focused on the effect of collaboration in creating a collective working memory among the members of a group. Previous research studies seldom included controlled randomized experiments and assessed learners’ writing products as a means to evaluate the effectiveness of collaborative learning. Therefore, according to the collective working memory effect, the reported experiment was designed to test the hypotheses that learners of English as a foreign language in the collaborative process-genre instructional condition would achieve better individual learning outcomes in terms of writing skills, experience lower levels of cognitive load, and have higher instructional efficiency than learners in the individual process-genre instructional condition.

## Materials and Methods

### Participants

The study adopted a purposive convenience sampling method; 64 undergraduate students (29 females) voluntarily participated in this experiment after reading the recruitment notice. They studied at a technological university in Shandong Province, China. They were also briefed about the aims, the procedures, their rights through the study, and their rights to access the research results. They were requested to return the signed consent form if they determined to participate. These college students were on average 21.5 years old and had spent 11 years learning English as a foreign language at the time of the experiment, so they could be regarded as having an intermediate level of English proficiency. They were randomly allocated into the individual learning condition (IL) (*n* = 32) and the collaborative learning condition (CL) (*n* = 32). The participants in the collaborative learning condition were further randomly allocated into eight groups with four members in each. This arrangement was based on the rationale that groups consisting of no more than six members could maximize participation by all group members (Herner et al., 2002).

The participants were required to write an essay as a pretest. The design of the pretest was based on Task 2 of the writing section in *International English Language Test System (IELTS): General Training*. Two independent raters examined their writings by complying with the *IELTS writing band descriptors*. These raters were proficient IELTS tutors with experience in applying the band descriptors in evaluating IELTS essays. An independent samples *t*-test indicated that the pre-test scores of the IL group (*M* = 5.16, *SD* = 0.91) were not significantly different from the CL group (*M* = 5.00, *SD* = 1.02), *t*(58) = 0.61, *p* > 0.05.

### Materials

The instructional material was about how to write complaint letters. The development of the teaching material was based on the book *The Official Cambridge Guide to IELTS* authored by Cullen et al. (2014). The experimental materials included four teaching components (structural features teaching, language features teaching, model essay teaching, and essay planning teaching), one essay planning phase, one testing phase (essay writing), and one subjective cognitive load rating phase (Appendix).

### Procedures

**FIGURE 1:**
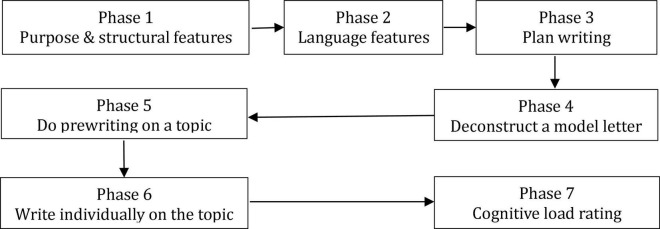
Seven phases of the research study.

The instruction was delivered in seven phases (see Figure 1). The participants in the individual learning condition were allocated to a lecture room. Each participant sat with at least 1-m distance from other participants to prevent collaboration and interference. The 84-member CL instructional groups were put in one lecture room. Each group kept a distance of at least 5 m from other groups to prevent collaboration and interference between groups, if any. The participants in the IL condition were required to complete all the seven phases individually; on the other hand, the participants in the CL condition completed the first five learning phases collaboratively, but the last two phases were completed independently. Associated questions were provided for thinking (for individual learners) and discussing (for collaborative learners) as Proske and Kapp (2013) argued that “learning questions might also be suitable to support the construction of a richly interconnected situation model of a writing topic which in turn may allow writers to produce better text products” (p. 1340). As it was generally believed that cognitive activities involved in writing procedures were recursive and dynamic (Flower and Hayes, 1981), the participants were reminded that they did not necessarily treat the phases as absolute linear orders and had the freedom to revisit the previous phase or skip to next one when they feel necessary.

The first part of the instructional materials (10 min) introduced the purposes and structural features of complaint letters, as well as the functions of each structural component. The structural features of complaint letters covered in this study include the following: the introductory paragraph elicits the purpose of complaint letters; the body paragraphs elaborate on the problems that letters are about and the suggested solutions; and the conclusion paragraph generally states the expectations and closes the letter. The associated questions for thinking (for individual learners) and discussion (for collaborative learners) were: *How do structural features reflect communicative purposes?* and *Are there alternative structures for this genre?* The second part (10 min) elaborated on the common language features of complaint letters, such as phrases and sentence structures for specifying the problem, outlining the consequences, making and justifying a specific claim, and so on, with the questions for thinking and discussion being: *Are there alternative ways to give reasons and solutions?* By using graphic organizers, the third part (10 min) showed the essential steps in planning writing. The question for introspection and discussion in this phase was: *If there exist alternative structures, how can these steps in essay planning be adapted to suit those structures?* The fourth part (15 min) introduced a model letter, in which the participants were required to identify the structural features, explain the functions of each feature, and the language features that were used for achieving the purposes. The associated questions in this phase were: *What tenses have been used mainly in each paragraph?* and *Why tenses were used in these ways?* The fifth instructional phase (10 min) required the participants to plan a letter on a given topic and scenario. In these five phases, the participants in the collaborative instructional conditions were encouraged to learn the materials through collaboration, share their understandings, ask questions, and provide responses, while the individual learners were encouraged to talk to themselves or engage in an internal conversation. In the sixth phase (15 min), the participants were required to individually write a letter on the topic they discussed in the fifth phase by using the skills learned in the first four phases. The last phase of the experiment (5 min) was a subjective cognitive load rating questionnaire (Appendix).

Traditionally, subjective ratings of working memory load have proven to be able to collect reliable and valid estimations of mental load in a non-intrusive way (Jiang and Kalyuga, 2020). The cognitive load rating questionnaire was developed from the questionnaires designed by Leppink et al. (2013), with the first six items on intrinsic cognitive load and the last six items on extraneous cognitive load. The questionnaire was written in Chinese, the research participants’ first language. The participants were asked to evaluate the appropriateness of a certain aspect of the instructional design that could orchestrate their mental resources to facilitate learning by choosing a number on a Likert-type scale, ranging from 0 (not at all the case) to 10 (completely the case). In addition, the instructor was available to clarify and explain puzzles and queries, if any.

### Scoring

The quality of the letters was assessed according to the *IELTS General Training Writing Task 1: Writing band descriptors* published by the British Council. The band descriptors cover four categories: task achievement, coherence and cohesion, lexical resource, and grammatical range and accuracy. Each category has the 9-point scale, ranging from one to nine. Each letter was given one score for each category, and the sum of the scores in the four categories was the rater’s score for the letter. The highest mark for a letter was 36. Two independent raters assessed students’ letters. The average value of two raters’ markings was used as the final score of the letter. The inter-rater reliability was calculated using a Person intra-class correlation (ICC). The ICC measure of 0.92 indicated a high degree of inter-rater reliability.

## Results

Table 1 shows means and standard deviations of the letter scores, the scores of each category, the ratings of intrinsic, extraneous, and overall cognitive load, and the instructional efficiency for the two instructional conditions. The reliability of the subjective cognitive load rating scale as measured by Cronbach’s alpha was 0.76.

**TABLE 1:** Means and standard deviations for essay writing performance scores, individual category score, subjective ratings of cognitive load, and instructional efficiency for two instructional groups.

Group		Individual learning (*N* = 32)	Collaborative learning (*N* = 32)
Essay score	*M*	22.03	24.55
	*SD*	4.36	4.21
Task achievement	*M*	5.72	6.19
	*SD*	1.08	1.05
Coherence and cohesion	*M*	5.44	6.14
	*SD*	1.07	1.06
Lexical resource	*M*	5.50	6.03
	*SD*	1.15	1.07
Grammatical range and accuracy	*M*	5.36	6.19
	*SD*	1.13	1.08
Intrinsic load	*M*	2.21	1.59
	*SD*	0.99	0.99
Extraneous load	*M*	2.40	1.97
	*SD*	0.68	0.75
Overall load	*M*	2.30	1.78
	*SD*	0.61	0.69
Efficiency	*M*	−0.47	0.46
	*SD*	1.02	1.08

An analysis of covariance (ANCOVA) was conducted to compare the two instructional groups’ letter scores, scores of each subcategory, the ratings of intrinsic cognitive load, extraneous cognitive load, and overall cognitive load, as well as the indicators of instructional efficiency. Levene’s test was conducted (*p* > 0.05) and the assumptions were satisfied. After controlling for the effect of pretest, it was found that the participants in the CL instructional condition demonstrated significantly better letter writing performance [*F*(1, 61) = 27.40, *p* = 0.001, *partialη*^2^ = 0.31] and significantly higher instructional efficiency [*F*(1, 61) = 31.97, *p* = 0.001, *partialη*^2^ = 0.34] than those in the IL instructional condition. In terms of category scores, the learners in the CL teaching condition significantly outperformed those learners in the IL teaching condition in all the four subscales: task achievement [*F*(1, 61) = 15.72, *p* = 0.001, *partialη*^2^ = 0.21], coherence and cohesion [*F*(1, 61) = 30.64, *p* = 0.001, *partialη*^2^ = 0.33], lexical resource [*F*(1, 61) = 17.86, *p* = 0.001, *partialη*^2^ = 0.23], as well as grammatical range and accuracy [*F*(1, 61) = 41.76, *p* = 0.001, *d* = 0.41]. The participants in the IL instructional condition experienced significantly higher levels of intrinsic cognitive load [*F*(1, 61) = 7.68, *p* = 0.007, *partialη*^2^ = 0.11], significantly higher levels of extraneous cognitive load [*F*(1, 61) = 5.83, *p* = 0.020, *partialη*^2^ = 0.09], and significantly higher levels of overall cognitive load [*F*(1, 61) = 12.02, *p* = 0.001, *partialη*^2^ = 0.17] than the participants in the CL condition.

The covariate, which is pretest in the study, was significantly related to the letter writing performance, which means that the participants in the CL condition had significantly better performance than the students in the IL condition in terms of the overall scores [*F*(1, 61) = 143.44, *p* = 0.001, *r* = 0.84] as well as the four subscales: task achievement [*F*(1, 61) = 127.86, *p* = 0.001, *r* = 0.81], coherence and cohesion [*F*(1, 61) = 128.09, *p* = 0.001, *r* = 0.82], lexical resource [*F*(1, 61) = 125.52, *p* = 0.001, *r* = 0.82], and grammatical range and accuracy [*F*(1,61) = 146.29, *p* = 0.001, *r* = 0.84]. In addition, cognitive load ratings and instructional efficiency were related to the covariate, pretest. The correlation to the covariate, pretests, was also observed in intrinsic cognitive load, overall cognitive load, and instructional efficiency. Students in the CL instructional condition had lower cognitive load ratings and higher instructional efficiency than the participants in the IL condition: intrinsic cognitive load [*F*(1, 61) = 5.49, *p* = 0.02, *r* = 0.28], overall cognitive load [*F*(1, 61) = 4.58, *p* = 0.036, *r* = 0.07], and instructional efficiency [*F*(1, 61) = 62.88, *p* = 0.001, *r* = 0.51]. However, it should be noted that the covariate, pretest, was not significantly related to extraneous cognitive load [*F*(1, 61) = 0.42, *p* > 0.05], which indicates that the differences in participants’ perception of extraneous cognitive load could be largely attributed to the dependent variable, instructional conditions.

## Discussion

The reported experiment was conducted to test the hypotheses generated by cognitive load theory that learners of English as a foreign language in a collaborative instructional condition would show better writing performance, lower levels of cognitive load, and higher instructional efficiency than learners in an individual learning condition. Even though relations to the covariate, pretest, were observed, the results of the study generally supported the hypotheses. As for the first hypothesis, this randomized experimental study found that the students in the collaborative learning condition demonstrated higher overall post-test letter writing scores and higher subcategory scores (task achievement, coherence and cohesion, lexical resource, as well as grammatical range and accuracy) than the participants in the individual learning condition. The second research hypothesis was also supported as the participants in the collaborative learning condition indicated lower overall cognitive load ratings than the participants in the individual learning condition. It was also found that the collaborative learning condition generated higher instructional efficiency in terms of developing writing skills than the individual learning condition. Moderate and significant negative correlations were found between the ratings of intrinsic cognitive load and the letter-writing performance scores for both instructional conditions. The results demonstrated the collective working memory effect (Kirschner et al., 2009, 2018; Sweller et al., 2011) in the domain of learning writing skills by learners of English as a foreign language. As predicted by cognitive load theory, in the case of complex learning tasks such as writing in a foreign language, the benefits of collective working memory exceeded the possible disadvantages of dealing with transaction costs involved in coordinating individual working memories.

First, the study contributed to the research area of writing in a foreign language by conceptualizing the research and interpreting the findings from the perspective of cognitive load theory. In an attempt to account for the role of specific cognitive mechanisms in improving writing performance, it is possible to assume that the collaborative instructional approach had created an effective pool of knowledge about language and a pool of cognitive resources that beneficially influenced the quality of written products (Storch, 2005; Strobl, 2014). The interactions in collaborative instructional conditions could trigger more learning-relevant cognitive mechanisms, for example, knowledge elaboration and internalization which are essential for meaningful and effective learning. These learning mechanisms could enable learners to organize information into ordered structures and integrate new information with prior knowledge structures (Dillenbourg, 1999; Kalyuga, 2009). In the process of collaborative learning, theme-related knowledge structures would be retrieved from learners’ long-term memory and function collectively as distributed cognition including “internal minds, external representations, and interactions among individuals” (Klein and Leacock, 2011, p. 133). The distributed cognition could evolve through members’ contributions using stating claims, supporting or challenging others’ opinions, providing supporting details, and so on. The mental activities in sharing, understanding, and negotiating meaning involve expressive or introspective elaborations, resulting in conceptual changes in group members (Dillenbourg, 1999). As more sources of information come to the group memory, learners would exercise more knowledge elaborations to establish links between new information and the existing knowledge structures, leading to better performance measures. The multiple learning phases in the collaborative conditions offered collaborators more opportunities to use the language-related episodes (LRE) and task-related episodes, which were supposed to benefit their writing.

Second, the findings are consistent with the collective working memory effect, in that learning English as a foreign language writing skills in the collaborative instructional condition is more effective and efficient than in the individual learning condition (Kirschner et al., 2009; Retnowati et al., 2018). As learning tasks used for teaching English as a foreign language writing skills are high in element interactivity, and multiple factors (such as linguistic and situational knowledge, understanding of audience and purposes, etc.) affect the learning process, it can be assumed that the participants in each collaborative group would provide collective scaffolding, resulting in learning more sophisticated writing skills in terms of lexical accuracy, grammatical complexity, logic organization, and so on, in the learning phases and consequently in the better performance of these learners in the testing phase than the participants in the individual learning condition.

In addition, this study also indicates that adopting a process-genre approach in a collaborative condition could lead to significantly better writing performance than in an individual learning condition, which is particularly consistent with research studies on developing self-regulation of writing processes and generic knowledge through collaborations (e.g., Graham and Sandmel, 2011; Jones, 2014; Wette, 2017; Villarreal and Gil-Sarratea, 2019; Teng, 2020). According to the genre approach to teaching writing skills, effective instructional practices should “offer writers an explicit understanding of how texts in target genres are structured,” teach “the lexico-grammatical patterns which typically occur in its different stages,” and cultivate writers to command “an awareness of target genres and an explicit grammar of linguistic choices” (Hyland, 2003b, p. 26). However, if all lexical, syntactical, structural, and logical contents were taught without appropriate sequencing and prioritizing, high levels of cognitive load could be generated. Therefore, segmenting a learning task into several phases can ameliorate the complexity of information as the number of interacting elements would be reduced. For example, in a controlled randomized experiment, Klein and Ehrhardt (2015) found that organizing instructional tasks into manageable parts helped learners generate more balanced claims and reduced high-achieving students’ cognitive load in writing persuasion texts as measured by the perceived difficulty of their learning.

Furthermore, the results of the reported study are also consistent with previous research in the field of collaborative learning of writing skills (e.g., Shehadeh, 2011; McDonough et al., 2018), in that the learners in the collaborative instructional condition had better qualities of prewriting/writing performance than the learners in the individual instructional condition. Still, this study contributed to the area of collaborative writing research in two novel ways. First, differently from most of the previous research which required all learners in a collaborative group to write a common single text, this study required every member in a collaborative condition to write a separate text, and the quality of individual texts was assessed to compare the effectiveness of individual and collaborative learning conditions on the same grounds. This method of measuring learning gains by assessing the quality of individual writing products is more valid and reliable according to Kirschner et al. (2009), as it better fits the learning goals. Second, the use of subjective ratings of participants’ cognitive load in learning and the calculation of instructional efficiency provided additional evidence to support a cognitive load interpretation of the results as the case of the collective working memory effect.

The reported study still has some limitations that require further research. First, this study did not consider the foreign language proficiency of the participants as a variable in collaborative teaching of English as a foreign language writing skill. According to the expertise reversal effect in cognitive load theory, the effectiveness of specific instructional techniques and procedures depends on the levels of the learner’s prior knowledge in the domain (Kalyuga et al., 2003; Kalyuga, 2007; Sweller et al., 2011). This effect has been demonstrated with all other instructional methods developed by cognitive load theory. It is likely that this effect also applies to the collective working memory effect. For example, Storch (2011) claimed that second language proficiency should be taken into consideration in implementing collaborative learning of writing skills. Therefore, future research studies may need to recruit learners at different proficiency (prior knowledge) levels to investigate possible interactions between levels of learner expertise in the area of English as a foreign language writing skills and the effectiveness of individual versus collaborative learning conditions. Second, this study examined the effectiveness of learning approaches (individual or collaborative) by primarily assessing the quality of learning products (i.e., essay). Future studies need to consider and measure other possible contributing factors and performance indicators, such as interactions in the writing processes, the quality of jointly drafted essays, and learners’ perceptions. In addition, as a way to manifest how collaborative learning affects the development of collective memory, future research should record and analyze learners’ interactions during the collaborative learning phases. Furthermore, more research should be done to investigate how learners develop their writing skills in other genres (such as argumentative, informative, and descriptive ones) in individual and collaborative instructional conditions.

In conclusion, the results of the reported experimental study supported the hypothesis generated by cognitive load theory. Learning English as a foreign language writing skills through a process-genre approach in the collaborative instructional condition was more effective and efficient than in the individual instructional condition. Subjective ratings of the cognitive load supported the interpretation of results within a cognitive load framework. The findings have implications for the innovations of teaching approaches, the developments of course materials, and curriculum designs in the field of teaching foreign language writing skills.

## Data Availability Statement

The raw data supporting the conclusions of this article will be made available by the authors, without undue reservation.

## Ethics Statement

Ethical review and approval was not required for the study on human participants in accordance with the local legislation and institutional requirements. The patients/participants provided their written informed consent to participate in this study.

## Author Contributions

DJ: conceptualization, methodology, resources, data curation, writing – original draft, and review and editing. SK: supervision and writing – review and editing. Both authors contributed to the article and approved the submitted version.

## Conflict of Interest

The authors declare that the research was conducted in the absence of any commercial or financial relationships that could be construed as a potential conflict of interest.

## Publisher’s Note

All claims expressed in this article are solely those of the authors and do not necessarily represent those of their affiliated organizations, or those of the publisher, the editors and the reviewers. Any product that may be evaluated in this article, or claim that may be made by its manufacturer, is not guaranteed or endorsed by the publisher.

## Appendix

### Subjective Rating of Cognitive Load

All of the following questions refer to the learning activity that you just finished. Please respond to each of the questions on the following scale (0 meaning not at all the case and 10 meaning completely the case).

**Table:**

No.	Question	Scale 0 = not at all the case; 10 = completely the case										
➀	The topic/topics covered in the activity was/were very complex.	0	1	2	3	4	5	6	7	8	9	10
➁	The activity covered generic structure that I perceived as very complex.	0	1	2	3	4	5	6	7	8	9	10
➂	The activity covered language features that I perceived as very complex.	0	1	2	3	4	5	6	7	8	9	10
➃	The activity covered writing planning that I perceived as very complex.	0	1	2	3	4	5	6	7	8	9	10
➄	The activity covered model essay that I perceived as very complex.	0	1	2	3	4	5	6	7	8	9	10
➅	The activity covered brainstorming that I perceived as very complex.	0	1	2	3	4	5	6	7	8	9	10
➆	The instructions and/or explanations were, in terms of learning, very ineffective.	0	1	2	3	4	5	6	7	8	9	10
➇	The instructions and/or explanations on generic structure during the activity were very unclear.	0	1	2	3	4	5	6	7	8	9	10
➈	The instructions and/or explanations on language features during the activity were very unclear.	0	1	2	3	4	5	6	7	8	9	10
➉	The instructions and/or explanations on writing planning during the activity were very unclear.	0	1	2	3	4	5	6	7	8	9	10
⑪	The instructions and/or explanations on model essay planning during the activity were very unclear.	0	1	2	3	4	5	6	7	8	9	10
⑫	The instructions and/or explanations on brainstorming during the activity were very unclear.	0	1	2	3	4	5	6	7	8	9	10
